# Zeit, dass sich was dreht: Was muss der qualitativ hochwertige chirurgische Weiterbildungsverbund von morgen bieten? Perspektiven und Forderungen der Jungen Foren der chirurgischen Fachgesellschaften Deutschlands

**DOI:** 10.1007/s00104-025-02269-0

**Published:** 2025-03-17

**Authors:** Frederik Schlottmann, Sebastian Schaaf, Romina Maria Rösch, Maria E. Dey Hazra, Marit Herbolzheimer, Sabine Drossard, Louisa Schuffert, Benedikt J. Braun, Sarah Lif Keller, Arash Motekallemi, Joscha Mulorz, Hruy Menghesha, Anna Lawson McLean, Tobias Huber, Frederic Bouffleur, Gerrit Freund

**Affiliations:** 1 Klinik für Plastische, Ästhetische, Hand- und Wiederherstellungschirurgie, Medizinische Hochschule Hannover, Junges Forum der Deutschen Gesellschaft für Plastische, Rekonstruktive und Ästhetische Chirurgie (DGPRÄC), Carl-Neuberg-Str. 1, 30625 Hannover, Deutschland; 2 Klinik für Allgemein‑, Viszeral- und Thoraxchirurgie, Bundeswehrzentralkrankenhaus Koblenz, Perspektivforum Junge Chirurgie der Deutschen Gesellschaft für Chirurgie (DGCH) und Chirurgische Arbeitsgemeinschaft Junge Chirurgie (CAJC) der Deutschen Gesellschaft für Allgemein- und Viszeralchirurgie (DGAV), Rübenacher Str. 170, 56072 Koblenz, Deutschland; 3 Klinik für Thoraxchirurgie und Translational Lung Research Center Heidelberg (TLRC), Thoraxklinik Heidelberg, Universitätsklinikum Heidelberg, Junges Forum der Deutschen Gesellschaft für Thoraxchirurgie (DGT), Röntgenstraße 1, 69126 Heidelberg, Deutschland; 4 Orthopädische Privatpraxis Dr. Kuhlee, Junges Forum O und U der Deutschen Gesellschaft für Orthopädie und Unfallchirurgie (DGOU) und des Berufsverbands für Orthopädie und Unfallchirurgie (BVOU), Hönower Str. 16, 12623 Berlin, Deutschland; 5 BG Unfallklinik Murnau, Junges Forum O und U der Deutsche Gesellschaft für Orthopädie und Unfallchirurgie (DGOU) und des Berufsverbands für Orthopädie und Unfallchirurgie (BVOU), Professor-Küntscher-Straße 8, 82418 Murnau am Staffelsee, Deutschland; 6 Klinik und Poliklinik für Allgemein‑, Viszeral‑, Transplantations‑, Gefäß- und Kinderchirurgie, Universitätsklinikum Würzburg, Arbeitskreis kinderchirurgischer Assistent*innen der Deutschen Gesellschaft für Kinder- und Jugendchirurgie (DGKJCH), Oberdürrbacher Str. 6, 97080 Würzburg, Deutschland; 7 Klinik und Poliklinik für Kinderchirurgie, Universitätsmedizin der Johannes Gutenberg-Universität Mainz, Arbeitskreis kinderchirurgischer Assistent*innen der Deutschen Gesellschaft für Kinder- und Jugendchirurgie (DGKJCH), Langenbeckstr. 1, 55131 Mainz, Deutschland; 8 Klinik für Unfall- und Wiederherstellungschirurgie an der Eberhard Karls Universität Tübingen, BG Klinik Tübingen, Themenreferat Nachwuchs, Berufsverband der Deutschen Chirurgie (BDC), Schnarrenbergstr. 95, 72076 Tübingen, Deutschland; 9 Unfall‑, Hand- und Orthopädische Chirurgie, Städtisches Klinikum Karlsruhe, Junges Forum O und U der Deutsche Gesellschaft für Orthopädie und Unfallchirurgie (DGOU) und des Berufsverbands für Orthopädie und Unfallchirurgie (BVOU), Moltkestraße 90, 76133 Karlsruhe, Deutschland; 10 Klinik für Herzchirurgie, Klinikum Kassel, Junges Forum der Deutschen Gesellschaft für Thorax‑, Herz- und Gefäßchirurgie (DGTHG), Mönchebergstraße 41–43, 34125 Kassel, Deutschland; 11 Klinik für Gefäß- und Endovaskularchirurgie, Universitötsklinikum Düsseldorf, Junges Forum der Deutschen Gesellschaft für Gefäßchirurgie und Gefäßmedizin (DGG), Moorenstraße 5, 40225 Düsseldorf, Deutschland; 12 Klinik für Allgemein‑, Viszeral‑, Thorax- und Gefäßchirurgie, Universitätsklinikum Bonn, Junges Forum der Deutschen Gesellschaft für Thoraxchirurgie (DGT), Venusberg-Campus 1, 53127 Bonn, Deutschland; 13 Klinik für Thoraxchirurgie, Helios Klinikum Bonn/Rhein-Sieg, Junges Forum der Deutschen Gesellschaft für Thoraxchirurgie (DGT), Von-Hompesch-Str. 1, 53123 Bonn, Deutschland; 14 Klinik und Poliklinik für Neurochirurgie, Universitätsklinikum Jena, Junges Forum der Deutschen Gesellschaft für Neurochirurgie (DGNC), Am Klinikum 1, 07747 Jena, Deutschland; 15 Klinik für Allgemein‑, Viszeral- und Transplantationschirurgie, Universitätsmedizin Mainz, Chirurgische Arbeitsgemeinschaft Junge Chirurgie (CAJC) der Deutschen Gesellschaft für Allgemein- und Viszeralchirurgie (DAV), Langenbeckstr. 1, 55131 Mainz, Deutschland; 16 Klinik für Mund‑, Kiefer- und Gesichtschirurgie, Universitätsklinikum Heidelberg, Junges Forum der Deutschen Gesellschaft für Mund‑, Kiefer- und Gesichtschirurgie (DGMKG), Im Neuenheimer Feld 400, 69120 Heidelberg, Deutschland; 17 Klinik für Plastische Chirurgie, Hand- und Verbrennungschirurgie, Universitätsklinikum Aachen, Junges Forum der Deutschen Gesellschaft für Plastische, Rekonstruktive und Ästhetische Chirurgie (DGPRÄC), Pauwelsstr., 52074 Aachen, Deutschland

**Keywords:** Weiterbildung, Medizinische Ausbildung, Gesundheitsreform, Ambulantes Operieren, KHVVG, Internship and residency, Medical education, Healthcare reform, Outpatient surgical procedures, Hospital Treatment Remuneration Improvement Act

## Abstract

**Hintergrund:**

Die Krankenhausstrukturreform durch das Krankenhausversorgungsverbesserungsgesetz (KHVVG) verändert ab 2025 die medizinische Versorgungslandschaft in Deutschland grundlegend. Ambulantisierung und sektorenübergreifende Versorgungsmodelle werden die chirurgische Weiterbildung erheblich beeinflussen. Insbesondere Weiterbildungsverbünde zwischen Kliniken und ambulanten Einrichtungen werden als zentrale Lösung diskutiert, um die chirurgische Weiterbildung zu sichern.

**Fragestellung:**

Ziel des vorliegenden Positionspapieres ist es, die Wünsche und Forderungen des chirurgischen Nachwuchses an eine Verbundweiterbildung zu formulieren. Diese sollen den beteiligten Akteurinnen und Akteuren als Leitfaden für eine erfolgreiche Umsetzung dienen.

**Material und Methoden:**

Das Positionspapier basiert auf Diskussionen der Jungen Foren chirurgischer Fachgesellschaften und Berufsverbände, Analysen aktueller Weiterbildungsmodelle und Erfahrungen aus Pilotprojekten. Es beschreibt Maßnahmen, die eine sektorenübergreifende, strukturierte Weiterbildung mit klaren rechtlichen, finanziellen und didaktischen Rahmenbedingungen fördern.

**Ergebnisse:**

Erfolgreiche Modelle, wie die Rotation in ambulante Einrichtungen, zeigen positive Effekte auf Weiterbildung und Praxiserfahrung. Notwendige Schritte umfassen arbeitsrechtliche Vereinfachungen, transparente Curricula, eine adäquate und verlässliche Finanzierung sowie eine zertifizierte didaktische Qualifikationen der Weiterbildenden. Pilotprojekte unterstreichen das Potenzial intersektoraler Kooperationen, während unzureichende rechtliche und finanzielle Strukturen als Haupthindernisse identifiziert werden.

**Schlussfolgerungen:**

Die Einführung verbindlicher Weiterbildungsverbünde ist essenziell, um die chirurgische Versorgung und Weiterbildung zu sichern. Neben politischem Handeln zur Finanzierung und Anpassung der Rahmenbedingungen wird die Initiative von Kliniken und ambulanten Einrichtungen benötigt. Langfristig sollte die chirurgische Weiterbildung in Deutschland ein Exzellenzmerkmal werden.

## Abstract (en)

**Background:**

The hospital structural reform through the Hospital Treatment Remuneration Improvement Act (Krankenhausversorgungsverbesserungsgesetz, KHVVG) will fundamentally change the medical care landscape in Germany starting in 2025. Outpatient care and cross-sectoral care models will substantially influence advanced surgical training. In particular, comprehensive training networks between hospitals and outpatient facilities are being discussed as a core solution to ensure surgical training.

**Objective:**

The aim of this position paper is to highlight the wishes and demands of young surgeons regarding training alliances. These should serve as guidelines for the stakeholders involved for a successful implementation.

**Material and methods:**

The position paper is based on discussions of the Young Forums of surgical societies, analyses of current further training models and experiences from pilot projects. It describes measures that promote cross-sectoral structured further training with clear legal, financial and didactic framework conditions.

**Results:**

Successful models, such as rotation in outpatient facilities, show a positive effect on further training and practical experience. Necessary steps include simplifications of labor legislation, transparent curricula, adequate financing and certified didactic qualifications for those providing surgical residency training. Pilot projects underline the potential of intersectoral cooperation, while inadequate legal and financial structures have been identified as the main obstacles.

**Conclusion:**

The introduction of mandatory continuous education networks is essential to ensure surgical residency training and improving the quality of surgical patient care. In addition to political action to finance and adapt the framework conditions, hospitals and outpatient facilities need to take the initiative. In the long term surgical continuing education in Germany should be of exceptional quality.

Mit der Verabschiedung des Krankenhausversorgungsverbesserungsgesetzes durch den Bundesrat im November 2024 ist ein tiefgreifender Wandel der ambulanten und stationären Versorgungsstruktur in den kommenden Jahren beschlossen worden. Neben umfassenden Änderungen der Rahmenbedingungen der chirurgischen Versorgung wird das Reformpaket auch die chirurgische Weiterbildung beeinflussen. Die spezifischen Auswirkungen auf die Weiterbildung sind aber bis zum heutigen Tage noch nicht vollumfänglich absehbar und auch abhängig von der konkreten Ausgestaltung, dem Rahmen und der Umsetzung des Gesetzes. Fachgesellschaftsübergreifend existiert ein Konsens, dass die Schaffung von Weiterbildungsverbünden das unabdingbare Mittel ist, um die qualitativ hochwertige chirurgische Versorgung von morgen zu sichern.

## Hintergrund

Die chirurgische Weiterbildung in Deutschland steht vor dem Hintergrund der Krankenhausstrukturreform im Rahmen des Krankenhausversorgungsverbesserungsgesetzes (KHVVG) mehr denn je vor großen Herausforderungen [14]. Mit der Verabschiedung des KHVVG durch den Bundesrat am 22.11.2024 besteht nun Gewissheit, dass sich die medizinische Versorgungslandschaft in Deutschland ab 2025 sukzessive und radikal wandeln wird. Die sektorenübergreifende Versorgungsplanung, die zunehmende Ambulantisierung chirurgischer Eingriffe und die Schaffung von Leistungsgruppen werden substanziellen Einfluss auf die Krankenhauslandschaft und somit auf die Weiterbildung des chirurgischen Nachwuchses haben. Voraussichtlich werden nur noch wenige der neu aufzustellenden Kliniken und Institutionen die Bedingungen für eine volle Weiterbildungsbefugnis erfüllen. In verschiedenen Diskussionsrunden zeigte sich ein Konsens, dass die Schaffung strukturierter Weiterbildungsverbünde, auch „Verbundweiterbildung“ genannt, unumgänglich und politisch gewollt ist [7, 12, 14]. Ein Ziel ist hierbei die Schaffung zukunftsfähiger intersektoraler Weiterbildungsverbünde zwischen ambulanten Einrichtungen und Kliniken [7, 12, 14]. Aktuelle Pressemitteilungen und Zeitungsartikel zeigen, dass die Bemühungen der einzelnen Akteure bei den politischen Entscheidungsträgern zunehmend Gehör zu finden scheinen.

Um die Perspektive der Weiterzubildenden abzubilden, wurden in mehreren Diskussionsrunden von Vertreterinnen und Vertretern der Jungen Foren der chirurgischen Fachgesellschaften und Berufsverbände Deutschlands zentrale Anforderungen an die Verbundweiterbildung diskutiert und ein Konsens erarbeitet, welcher hier im Folgenden erläutert werden soll. Das vorliegende Positionspapier erörtert verschiedene Aspekte einer qualitativ hochwertigen Verbundweiterbildung und stellt einen Katalog an Maßnahmen vor, um die chirurgische Weiterbildung in Deutschland zukunftsfähig weiterzuentwickeln.

## Herausforderungen und Rahmenbedingungen für einen qualitativ hochwertigen Weiterbildungsverbund

### Notwendigkeit einer dynamischen sektorenübergreifenden Versorgung

Durch eine zunehmende Ambulantisierung wird ein erheblicher Anteil insbesondere kleinerer Eingriffe, welche für die Weiterbildung oft eine wichtige Rolle spielen, in den ambulanten Sektor verschoben und ist damit im stationären Setting nicht mehr abbildbar. Somit ist die Kooperation von Kliniken der Maximalversorgung mit Kliniken der Regelversorgung, medizinischen Versorgungszentren (MVZ) und Praxen zwingend erforderlich, um das volle Spektrum des jeweiligen chirurgischen Faches abzubilden. Laut Ärztestatistik 2022 sind derzeit nur 32 % der Chirurginnen und Chirurgen im ambulanten Bereich tätig, von denen nur ein Teil über eine Weiterbildungsermächtigung verfügt [2]. Es ist somit unumgänglich, die chirurgische Weiterbildung im ambulanten Sektor weiter auszubauen, um die entsprechenden ambulanten Weiterbildungsmöglichkeiten zu gewährleisten.

Ein Beispiel für die intersektorale chirurgische Weiterbildung im Rahmen eines Weiterbildungsverbundes liefert die Orthopädie und Unfallchirurgie in Kiel, das im November 2024 im Rahmen des Deutschen Krankenhaustags präsentiert wurde. In diesem Modell rotierten Weiterzubildende im 4. oder 5. Weiterbildungsjahr von der Klinik in ein Medizinisches Versorgungszentrum (MVZ), um dort Eingriffe durchzuführen, die im stationären Setting nur eingeschränkt möglich sind. Neben den wertvollen Einblicken in den ambulanten Sektor wurden auch die reduzierte Bürokratie und der geringere Anteil an Stationsarbeit als positive Begleiteffekte hervorgehoben. Die Erfahrungsberichte von Weiterbildenden und Weiterzubildenden waren in diesem Fall durchweg positiv [15].

Ähnliche Pilotprojekte sind auch in anderen Bundesländern auf regionaler Ebene etabliert. Es sollte jedoch berücksichtigt werden, dass in Ermangelung einer verbindlichen Verpflichtung zur Einführung von Weiterbildungsverbünden das Engagement einzelner Akteure auf regionaler Ebene von entscheidender Bedeutung ist. Diese Akteure sollten als Positivbeispiel mit Vorbildcharakter vorangehen. Gleichzeitig sollten die dort geschaffenen Strukturen zur unbürokratischen Umsetzung dieser Verbünde auch einen entscheidenden Präzedenzcharakter für Regionen haben, die einer solchen Struktur bisher weniger zugänglich waren.

### Verbindliche rechtliche und organisatorische Rahmenaspekte eines Weiterbildungsverbundes

Die Weiterbildung in der Chirurgie findet derzeit noch überwiegend im stationären Sektor statt. Aufgrund der politisch angestoßenen intersektoralen Dynamik wird dies zukünftig so nicht mehr abbildbar sein. Hospitationen in anderen Kliniken und Praxen werden aufgrund fehlender Kooperationen oder arbeitsrechtlicher Hürden bisher nicht regelhaft angeboten, sondern müssen durch die Weiterzubildenden eigenständig organisiert werden. Zudem sind Standortwechsel zwischen dem stationären und ambulanten Sektor aktuell fast immer mit einem Wechsel des Arbeitgebers oder Überlassungsvereinbarungen der Arbeitgeber untereinander verbunden. In den Jungen Foren der chirurgischen Fachgesellschaften und Berufsverbände herrscht Konsens, dass Standortwechsel unter bestimmten Voraussetzungen auch wohnortfern akzeptiert würden, wenn im Gegenzug eine qualitativ hochwertige chirurgische Weiterbildung erfolgt.

In einem qualitativ hochwertigen Weiterbildungsverbund müssen träger- und standortübergreifende Arbeitsverträge geschlossen werden, die alle Aspekte der intersektoralen Dynamik berücksichtigen. Nur so können sichere und verlässliche arbeitsrechtliche Rahmenbedingungen für Weiterzubildende und Arbeitgeber geschaffen werden. Wünschenswert ist, dass die Weiterzubildenden mit dem Weiterbildungsverbund einen Arbeitsvertrag bis zur Facharztprüfung abschließen und anschließend im Rahmen ihrer Weiterbildung anhand eines strukturierten Weiterbildungscurriculums durch die verschiedenen Standorte des Weiterbildungsverbundes sowohl im ambulanten als auch stationären Sektor rotieren. Entsprechende Musterarbeitsverträge sollten durch die Bundesärztekammer (BÄK) in Kooperation mit den beteiligten Tarifparteien und Fachgesellschaften erarbeitet werden. Bei der Ausgestaltung der Arbeitsverträge sollten insbesondere auch flexible Arbeitszeitmodelle mitgedacht werden. Der Arbeitsvertrag würde den Weiterzubildenden garantieren die erforderlichen Weiterbildungsabschnitte und -inhalte durchlaufen zu können, einhergehend mit einer Planungssicherheit für die Arbeitgeber.

### Adäquate Finanzierung von Weiterbildungsverbünden

Laut aktuellen Erhebungen des Deutschen Krankenhausinstitutes berichteten 65 % der befragten Kliniken im November 2023 von einer schlechten bis sehr schlechten Finanzierungslage [1]. Ein Trend, der sich für das Jahr 2024 noch verstärkt fortsetzte. Als Hauptproblem der ärztlichen Weiterbildung in Deutschland ist in einem vorherigen Positionspapier die fehlende finanzielle Abbildung der Weiterbildung identifiziert worden [1]. Im bisherigen klinischen Abrechnungssystem nach „disease related groups“ (DRG) oder „Gebührenordnung für Ärzte“ (GOÄ) findet die Weiterbildung derzeit keine Berücksichtigung. Die ärztliche Weiterbildung wird demnach seit der Einführung der DRG nicht finanziert. Dies ist aus Sicht der Jungen Foren der chirurgischen Fachgesellschaften inakzeptabel. Dadurch ist es für stationäre und ambulante Weiterbildungsstätten nach wie vor vordergründig nicht attraktiv, Zeit und Geld in die Ausbildung des chirurgischen Nachwuchses zu investieren. Da in den Kliniken der Facharztstandard gilt, werden Weiterzubildende hier häufig primär als kostengünstige Arbeitskräfte (aus)genutzt. In der ambulanten Versorgung gilt der Facharztstatus, hier sind Lösungen zu finden, die eine Erbringung der Leistungen durch Weiterzubildende ermöglicht. Zur Absicherung einer ausreichenden Finanzierung der ressourcenintensiven Weiterbildung wurden verschiedene Modelle diskutiert, von denen bisher keines im KHVVG berücksichtigt wird. Die Zusage einer Refinanzierung der ärztlichen Weiterbildung basiert derzeit auf öffentlichen politischen Erklärungen, denen jedoch bisher noch keine konkreten Maßnahmen gefolgt sind. Es ist unbestritten, dass ein funktionierender und qualitativ hochwertiger Weiterbildungsverbund nur dann realisierbar ist, wenn die finanzielle Grundlage sowohl in der Klinik als auch in der Praxis geklärt ist. Diese Finanzierung erachten wir als Kernpunkt der Forderung und letztlich als gesamtgesellschaftliche Verantwortung, da nur ein adäquat ausgebildeter Nachwuchs auch in Zukunft eine Versorgung auf einem für die Bevölkerung gewohnten hohen Niveau sicherstellen kann.

Nach erfolgter Regierungsbildung in der kommenden Legislaturperiode muss die Klärung der Finanzierung der ärztlichen Weiterbildung umgehend erfolgen, um die Zukunft der chirurgischen Weiterbildung und Versorgung nicht weiter zu schädigen. Der mögliche Lösungsansatz sollte dabei darauf abzielen, dass Weiterbildungsstätten für die angebotene und geleistete chirurgische Weiterbildung angemessen entlohnt und die entstandenen Kosten vollständig refinanziert werden. Dabei sollten auch die entstehenden Kosten für externe Kurs- und Fortbildungsangebote zur theoretischen und praktischen Qualifizierung berücksichtigt werden. Aktuell besteht die Möglichkeit zur Refinanzierung einer Weiterbildungsstelle in Praxis oder MVZ über § 75a SGB V mit einer derzeitigen Förderung in Höhe von 5400,00 € pro Monat durch die Kassenärztliche Vereinigung. Die Fördermöglichkeiten sind in der ambulanten Versorgung der Allgemeinmedizin, um die hausärztliche Versorgung zu sichern, bereits etabliert, in der ambulanten Chirurgie jedoch bisher nur selten anzutreffen. Somit sind die Mittel und damit auch die chirurgischen ambulanten Weiterbildungsstellen anders als in der Allgemeinmedizin begrenzt. Eine Kopplung der Refinanzierung an eine nachweislich qualitativ hochwertige Weiterbildung im Rahmen eines Weiterbildungsverbundes wäre ebenfalls eine denkbare Option.

### Didaktische Qualifikation im Weiterbildungsverbund

Auch wenn zunehmend eine kompetenzbasierte Weiterbildung und strukturierte Curricula gefordert werden, erfolgt die chirurgische Weiterbildung in Deutschland derzeit überwiegend arbeitsplatzbasiert und nicht strukturiert [4, 5]. Aktuelle Umfragen zeigen, dass der chirurgische Nachwuchs Optimierungsbedarf bei der derzeitigen angebotenen Weiterbildung sieht. So zeigte eine Umfrage in der Gefäßchirurgie, dass nur 39,5 % der Befragten mit ihrer Weiterbildung generell zufrieden waren, während 30,7 % angaben, mäßig zufrieden zu sein. Zudem gaben 53 % an, dass kein strukturiertes Weiterbildungskonzept an ihrem Standort existieren würde [3]. Dies deckt sich mit Zahlen aus der kinderchirurgischen Weiterbildung, in der 59 % der Teilnehmenden einer Umfrage angaben, kein strukturiertes Weiterbildungscurriculum zu haben (eigene unveröffentlichte Daten). Vergleichbare Daten zeigen sich auch in der Mund‑, Kiefer- und Gesichtschirurgie, wo dies von 46 % der Teilnehmenden einer Umfrage berichtet wurde [11]. Ähnliche Daten werden auch durch das Junge Forum Orthopädie und Unfallchirurgie sowie das Junge Forum der Deutschen Gesellschaft für Gefäßchirurgie und Gefäßmedizin berichtet [3, 6]. Des Weiteren zeigt die aktuelle Studienlage, dass der überwiegende Anteil der chirurgischen Weiterbildung nicht durch den Weiterbildungsermächtigen oder die Weiterbildungsermächtigte selbst erfolgt, sondern an andere Fachärztinnen und Fachärzte delegiert wird und häufig von diesen abhängig ist [10, 13]. Auch ein Personalschlüssel von Weiterbildenden zu Weiterzubildenden wird derzeit nicht vorgegeben.

Die Schaffung von Verbundweiterbildungen bietet die Möglichkeit, den bisherigen Problematiken zu begegnen und die chirurgische Weiterbildung didaktisch neu zu gestalten. Hier bedarf es der Ausarbeitung eines sektorenübergreifenden Weiterbildungscurriculums, in welchem transparent dargestellt wird, wann und wie die einzelnen Weiterbildungsinhalte über den gesamten 6‑jährigen Zeitraum der Weiterbildung vermittelt werden. Die im Laufe der Weiterbildung zu erreichenden Kompetenzen und Eingriffe sollten im Curriculum angepasst an Spektrum, Fallzahl und Qualifikation der entsprechenden Weiterbildungsstätte vorab festgelegt werden. Hierdurch können bereits bei der Planung der Rotationen mögliche Defizite aufgedeckt und gezielt individuelle Schwerpunkte gesetzt werden, um die notwendigen Kompetenzen nach Durchlaufen aller Weiterbildungsabschnitte zu erlangen. Eine enge Abstimmung der Weiterbildenden aus Klinik und Praxis ist dabei unerlässlich. Auch die Art und Weise der Wissensvermittlung mit modernen Lehr- und Lernkonzepten im Rahmen interner und externer Fortbildungen, Simulationsszenarien, Operationskursen, E‑Learning-Angeboten und Extended Reality sollte dabei definiert und berücksichtigt werden. Die Anwendung des Teilschrittekonzeptes zur eigenständigen Durchführung von Teilschritten eines chirurgischen Eingriffs sollte ebenfalls mit einbezogen werden [8, 9]. Zudem bedarf es einer zertifizierten didaktischen Qualifizierung der Weiterbildenden, um ihnen standardisiert das methodische Rüstzeug der Wissensvermittlung an die Hand zu geben. Hier sei exemplarisch auf die „Train-the-Trainer“-Kurse der Deutschen Gesellschaft für Chirurgie verwiesen, die ein entsprechendes Angebot abbilden. Es sind jedoch weitere flächendeckende und fachspezifische Angebote notwendig. Um die Qualität der Weiterbildung zu gewährleisten, sollten die Weiterbildungsermächtigten entsprechend ihrer Verantwortung für die Aus- und Weiterbildung anhand festgelegter, transparenter Qualitätskriterien ausgewählt sowie speziell geschult und laufend evaluiert werden. Der Exzellenzcharakter der chirurgischen Weiterbildung sollte auch im europäischen Vergleich vermehrt in den Fokus genommen werden. Statt als Bürde, die neben einem ausgelasteten, bürokratisch überladenen Klinikalltag noch absolviert werden muss, sollte Weiterbildung als Chance und Privileg begriffen werden.

### Einbeziehung der Bundes- und Landesärztekammern

Derzeit obliegt die Hoheit der Umsetzung der Musterweiterbildungsordnung (MWBO) der BÄK den 17 Landesärztekammern (LÄK). Nicht selten wird die MWBO dabei unterschiedlich ausgelegt, wodurch sich die Weiterzubildenden mit erheblichen bürokratischen Hürden konfrontiert sehen. Bei einem Wechsel der zuständigen LÄK im Rahmen eines Arbeitgeberwechsels gestaltet sich die Anerkennung von außerhalb des Zuständigkeitsbereichs erworbenen Weiterbildungsinhalten meist kompliziert und zieht lange Bearbeitungszeiträume nach sich. Eine einheitliche Umsetzung der MWBO durch die LÄK wäre hier wünschenswert und sollte durch entsprechende Anpassungen der MWBO vereinfacht werden.

Mit der Etablierung von Weiterbildungsverbünden wird es notwendig, dass auf regionaler Ebene nicht nur eine intersektorale Kooperation, sondern auch eine enge Zusammenarbeit über die Grenzen der Bundesländer und LÄK hinweg stattfindet. Daher erfordert es eine bundesland- und kammerübergreifende Anerkennung von Weiterbildungsinhalten, die im Rahmen eines Weiterbildungsverbundes erworben wurden. Diesbezüglich ist eine produktive Zusammenarbeit und enge Abstimmung der beteiligten LÄK untereinander, aber auch mit Weiterbildungsverbünden unabdingbar. In einer ersten Phase sollten insbesondere Pilotprojekte auf regionaler Ebene seitens der zuständigen LÄK beratend unterstützt und gefördert werden, um die gewonnenen Erkenntnisse und Erfahrungen für den weiteren zukunftsfähigen Ausbau von Weiterbildungsverbünden zu nutzen. Bei der inhaltlichen und didaktischen Ausgestaltung der Weiterbildungsverbünde sollten zudem die jeweiligen Fachgesellschaften beratend involviert werden.

#### Die Jungen Foren der chirurgischen Fachgesellschaften Deutschlands halten folgende Aspekte für essenziell, um einen qualitativ hochwertigen Weiterbildungsverbund zu realisieren

- Adäquate Finanzierung der chirurgischen Weiterbildung in Klinik und Praxis
- Verbindliche rechtliche Rahmenbedingungen für sektoren- und arbeitgeberübergreifende Arbeitsverträge
- Offenlegung der sektoren- und arbeitgeberübergreifenden Weiterbildungscurricula
- Kontinuierliches Monitoring und Evaluation von Weiterbildungsverbünden und dynamische Anpassung derselbigen
- Zertifizierte didaktische Qualifizierung von Weiterbildenden
- Anerkennung von Weiterbildungsverbünden und geleisteten Weiterbildungsinhalten landesärztekammerübergreifend
- Umfassende Unterstützung von Pilotprojekten durch LÄK und Fachgesellschaften

#### Dieses Positionspapier wird unterstützt von

- Perspektivforum Junge Chirurgie (PFJC) der Deutschen Gesellschaft für Chirurgie (DGCH)
- Junges Forum der Deutschen Gesellschaft für Plastische, Rekonstruktive und Ästhetische Chirurgie (DGPRÄC)
- Junges Forum O und U der Deutschen Gesellschaft für Orthopädie und Unfallchirurgie (DGOU) und des Berufsverbands für Orthopädie und Unfallchirurgie (BVOU)
- Chirurgische Arbeitsgemeinschaft Junge Chirurgie (CAJC) der Deutschen Gesellschaft für Allgemein- und Viszeralchirurgie (DGAV)
- Junges Forum der Deutschen Gesellschaft für Gefäßchirurgie und Gefäßmedizin (DGG)
- Arbeitskreis kinderchirurgischer Assistent*innen (AkA) der Deutschen Gesellschaft für Kinderchirurgie (DGKCH)
- Junges Forum der Deutschen Gesellschaft für Mund‑, Kiefer- und Gesichtschirurgie (DGMKG)
- Junges Forum der Deutschen Gesellschaft für Neurochirurgie (DGNC)
- Junges Forum der Deutschen Gesellschaft für Thoraxchirurgie (DGT)
- Junges Forum der Deutschen Gesellschaft für Thorax‑, Herz- und Gefäßchirurgie (DGTHG)
- Themenreferat Nachwuchs des Berufsverbandes der Deutschen Chirurgie e. V. (BDC)

## Fazit für die Praxis

Die Krankenhausreform wird erheblichen Einfluss auf die chirurgische Weiterbildung nehmen. Zum Erhalt der Qualität in der chirurgischen Versorgung und Weiterbildung des Nachwuchses bedarf es der Implementierung von Weiterbildungsverbünden, die das gesamte Spektrum des jeweiligen Fachgebietes abbilden und gleichzeitig einer klaren Finanzierung und Rotationsstruktur unterliegen. So lange es noch keine Verpflichtung zur Implementierung von Weiterbildungsverbünden gibt, kann nur an die Verantwortlichkeit und das Engagement der Zuständigen in Klinik und Praxis appelliert werden, um gemeinschaftlich Ideen und Konzepte für einen Weiterbildungsverbund zu entwickeln und zu etablieren. Zudem muss durch die Politik nach der erfolgten Bundestagswahl umgehend eine Klärung der Finanzierung sowie eine Anpassung der arbeits- und tarifrechtlichen Rahmenbedingungen erfolgen.

Die Jungen Foren der chirurgischen Fachgesellschaften Deutschlands fordern eine konstruktive Umsetzung des Themas „Verbundweiterbildung“ von allen beteiligen Akteurinnen und Akteuren zur Wahrung einer qualitativ hochwertigen chirurgischen Versorgung, da nur ein adäquat ausgebildeter Nachwuchs auch in Zukunft eine Versorgung auf einem für die Bevölkerung gewohnten hohen Niveau sicherstellen kann (Infobox 1).
